# Microfluidic Electroporation Coupling Pulses of Nanoseconds and Milliseconds to Facilitate Rapid Uptake and Enhanced Expression of DNA in Cell Therapy

**DOI:** 10.1038/s41598-020-63172-8

**Published:** 2020-04-08

**Authors:** An-Yi Chang, Xuan Liu, Hong Tian, Liping Hua, Zhaogang Yang, Shengnian Wang

**Affiliations:** 1 Chemical Engineering, Louisiana Tech University, PO Box 10137, Ruston, LA 71272 USA; 2 Institute for Micromanufacturing, Louisiana Tech University, PO Box 10137, Ruston, LA 71272 USA; 3 Macromolecular and Nanotechnology, Louisiana Tech University, PO Box 10137, Ruston, LA 71272 USA; 4 Center for Biomedical Engineering and Rehabilitations, Louisiana Tech University, PO Box 10137, Ruston, LA 71272 USA; 5 Department of Radiation Oncology, The University of Texas Southwestern Medical Center, Dallas, TX 75390 USA

**Keywords:** Transfection, Bionanoelectronics

## Abstract

Standard electroporation with pulses in milliseconds has been used as an effective tool to deliver drugs or genetic probes into cells, while irreversible electroporation with nanosecond pulses is explored to alter intracellular activities for pulse-induced apoptosis. A combination treatment, long nanosecond pulses followed by standard millisecond pulses, is adopted in this work to help facilitate DNA plasmids to cross both cell plasma membrane and nuclear membrane quickly to promote the transgene expression level and kinetics in both adherent and suspension cells. Nanosecond pulses with 400–800 ns duration are found effective on disrupting nuclear membrane to advance nuclear delivery of plasmid DNA. The additional microfluidic operation further helps suppress the negative impacts such as Joule heating and gas bubble evolution from common nanosecond pulse treatment that lead to high toxicity and/or ineffective transfection. Having appropriate order and little delay between the two types of treatment with different pulse duration is critical to guarantee the effectiveness: 2 folds or higher transfection efficiency enhancement and rapid transgene expression kinetics of GFP plasmids at no compromise of cell viability. The implementation of this new electroporation approach may benefit many biology studies and clinical practice that needs efficient delivery of exogenous probes.

## Introduction

Electroporation works by applying short, high-voltage electrical pulses across the cell membrane to make it transiently permeable to exogeneous cargos^1^. Although sub-microsecond charging time is enough to temporarily surpass the cell membrane capacitance, the actual pulse duration in standard electroporation practice is within sub-millisecond to millisecond range to allow effective payload delivery while ensure successful recovery of the integrity of cell plasma membrane and the survival of the treated cells. Nevertheless, owing to its balance of operation simplicity, transfection effectiveness, and broad allowance of probe or cell types, such reversible electroporation systems are often chosen as the preferred delivery approach in the past two decades to facilitate cellular internalization of plasmid DNA, oligonucleotides, and molecule drugs in many biological and clinical studies^2–4^. Besides such reversible electroporation treatment, ultra-sharp, but ultra-short electrical pulses (10–300 kV/cm with 10–300 ns pulse duration) were recently found useful to induce cell apoptosis for cancer treatment^5–10^. These nanosecond pulses are believed to alter the status of ions (e.g., Ca^2+^) and biomolecules (e.g., phosphatidylserine) around cell plasma membrane or other membrane structure of intracellular organelles, either temporarily or permanently^11–13^. These intracellular changes could greatly impact the signal pathways inside cells, leading to an increase of calcium concentration within cytosol, DNA fragmentation, caspase activation, and cell shrinkage^14–16^. Therefore, most early studies on nanosecond pulse treatments focus on enhancing those irreversible changes on organelles inside treated cells and their consequent apoptosis. There are a few attempts later that look into its potential for promotion of gene delivery since nanosecond pulses, in principle, could improve the permeability of cell nuclear membrane^17–19^. However, owing to its ultra-short pulse duration and ineffectiveness on breaking down the plasma membrane, disappointing gene transfection results are mostly found unless extremely high field strength was applied in some cases^20^. But this is often tied with low cell viability for severe side effects are associated with the applied high electrical voltage^21–25^. DNA transfection with nanosecond pulse treatment still faces substantial challenges to receive both desirable gene expression and cell viability.

**Figure 1:**
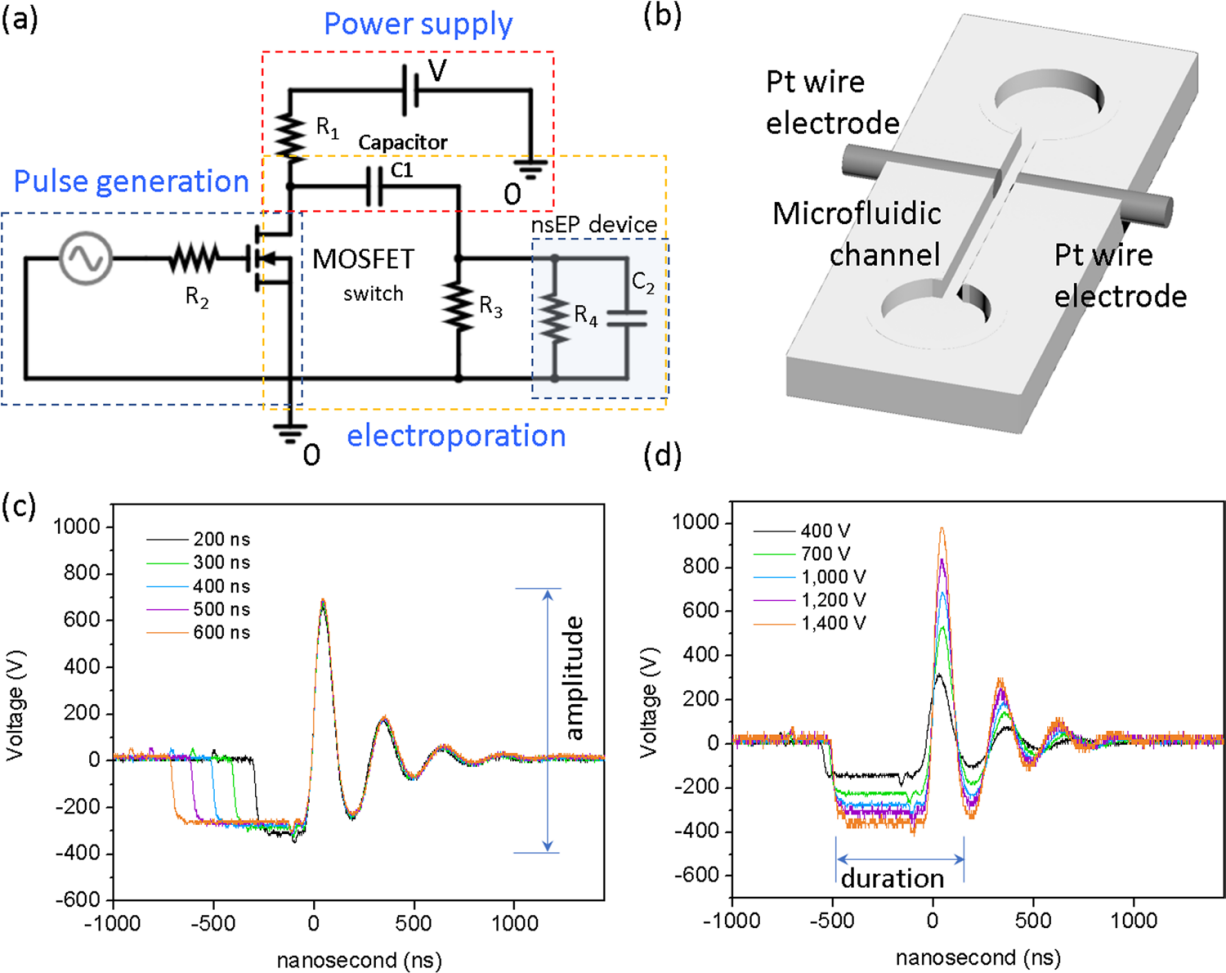
Nanosecond pulse electroporation (nsEP) system. (**a**) The electric circuit design; (**b**) the microfluidic configuration with Pt electrodes integrated on the channel wall from both sides; (**c,d**) the pulse profile of individual nanosecond electric pulses at different pulse widths (**c**) and pulse voltages (**d**).

Speeding up nuclear delivery of plasmids is still highly desired in DNA-based gene delivery as genetic probes were found to degrade very quickly even after cell internalization^26,27^. Unfortunately, available nuclear permeabilizing agents (e.g., *trans*-cyclohexane-1,2-diol or TCHD) are very toxic^28^. Considering its potential for disrupting the nuclear membrane structure^12,29^, we hypothesize that appropriate nanosecond pulse treatment might promote quick nuclear entry of plasmids from cell cytoplasm while microfluidics could help suppress its associated negative impacts on cells caused by the frequent application of high-voltage, nanosecond pulses. Through this work, we demonstrate this concept by coupling appropriate nanosecond pulse treatment (designated as “nsEP”) with traditional millisecond standard electroporation (designated as “msEP”) to allow cell uptake of plasmid quickly and efficiently. A microfluidic configuration is introduced during the nsEP treatment to effectively mitigate the side effects induced by electrohydrolysis and Joule heating which are commonly seen in other nanosecond pulse treatments. In our nsEP circuit, the nanosecond pulse generation unit is separated from the high-voltage supply unit while linked by a MOSFET switch (Fig. 1a). The rectangular signal from the pulse generator periodically triggers the closure of the electroporation circuit through the MOSFET switch to allocate high-voltage pulses with nanosecond duration. The nanosecond pulse parameters (i.e., voltage, duration, and frequency) can be flexibly regulated by adjusting the pulse generator signal. Two Pt wire electrodes are integrated on the side wall of a home-made microfluidic electroporation channel to help shorten the response time and lower the electroporation voltage (Fig. 1b). All these new features together enable stable pulse voltage and well-retained nanosecond pulse profile when tuning some pulse parameters in nsEP treatment (Fig. 1c,d). As some oscillations and reflections exist at the pulse profile tail, the pulse duration mentioned in this work is defined as the period from the primary rectangular pulse and the followed first spike-wave pulse, as illustrated in Fig. 1c,d.

In this new system, nanosecond pulses are first imposed on cells to make the nuclear membrane permeable, followed by a standard millisecond pulse treatment which breaks down the cell plasma membrane. This allows the targeting DNA probes to transport across both types of membrane barrier quickly to arrive in nucleus. Different from other nanosecond pulse studies with pulse durations of 60–300 ns^30^, we choose slightly longer pulses (400–800 ns) in our nsEP treatment so that the disruption of the nuclear membrane is more effective. Two separated pairs of electrodes are used: one pair of platinum wire-based electrodes serve in nanosecond pulse steps and another pair of standard parallel-plate aluminum electrodes in millisecond electroporation treatment. Such arrangements help avoid signal entanglement between nsEP and msEP treatment steps. The microfluidic configuration in our nsEP treatment also helps suppress the gas bubble evolution and consequent damage to cells and/or low cell viability that is often observed in electroporation with ultrashort pulses. The continuous flow-through operation further allows these treatments to be done to a large population of cells in high throughput. The new conceptual system was tested on both CT26 (an adherent cell representative) and K562 (a suspension cell representative) cells for DNA plasmid transfection. Demonstrated with quick kinetics and significantly improved transgene expression without compromise of cell viability, the success of this new electroporation system on these representative cell lines as transfection host or cancer study models may help advance many biology studies and clinical practice on cell function regulation and therapeutic effectiveness verification.

## Results

### Enhancement on reporter gene transfection

We hypothesis nsEP treatment is capable of disrupting the nuclear membrane. Therefore, a combination of nsEP and msEP treatment was carried out and compared to an alternative treatment in which a chemical treatment using TCHD and msEP was combined. TCHD is an amphipathic alcohol that is believed to reversibly collapse the permeability barrier of the nuclear pore complexes. It has been proven to facilitate the nuclear entry for plasmid DNA, though very toxic to the treated cells. Our comparison was made among three different cases: standard electroporation alone (designated as “msEP”), TCHD treatment followed by standard electroporation (designated as “TCHD + msEP”), and nanosecond pulse treatment followed by standard electroporation (designated as “nsEP+msEP”). The pulse conditions of msEP were briefly optimized and set as one pulse of 550 V/cm for pulse strength and 10 ms for pulse duration on both CT26 and K562 cells. The pulse conditions of nsEP were optimized on three parameters separately: pulse voltage, pulse duration, and pulse frequency. As shown in Supplemental Fig. S1, the best nsEP conditions with a balance of high transfection efficiency and reasonable cell viability are also similar for these two cell lines. For most nsEP tested - unless specified - the following nsEP conditions are adopted in later tests: 50 kV/cm (pulse strength), 600 ns (pulse duration), and 100 kHz (pulse frequency). Brief optimization of cell number or cell concentration in electroporation solution was also made with a fixed plasmid dosage to cell number ratio as 10 µg pMaxGFP DNA/10^6^ cells (Supplemental Fig. S2). A cell number of 10^6^ cells/100 µL or 10^7^ cells/mL, which is often seen in recommended protocols, was adopted in experiments of this work unless specified.

**Figure 2:**
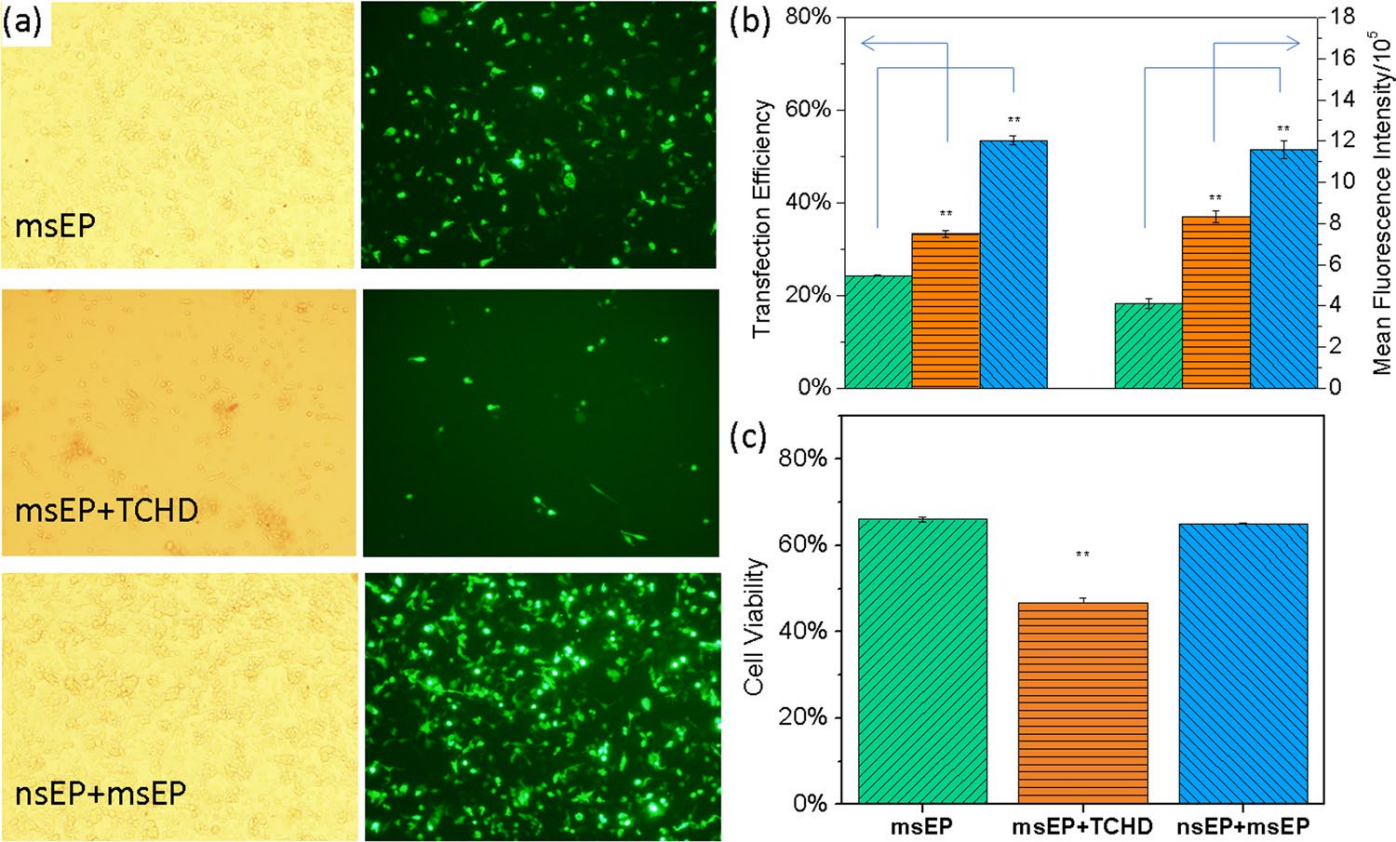
The transfection and cell viability of CT26 cells 24 hrs after transfection in three difference cell transfection methods with a cell concentration of 10^7^ cells/mL. (**a**) The phase contrast (left) and fluorescent (right) images of cells treated by each method; (**b**) quantitative transgene expression presented both by transfection efficiency as the number percentage of GFP positive cells (data on the left group) and mean fluorescence intensity for average GFP dosage in individual cells (data on the right group, readings were divided by a factor of 10^5^); (**c**) cell viability by MTS assays for cells treated by each method. In panels b and c, “msEP alone”: columns with green, upward diagonal stripes; “msEP+TCHD”: columns with orange, horizontal stripes; “nsEP+msEP”: columns with blue downward diagonal stripes. Significance t-test was performed with comparison to “msEP alone” treatment (n = 3) with **P < 0.01 and *** P < 0.005.

Under these pulse conditions, the “nsEP+msEP” treatement receives higher transfection efficiency and lower toxicity in adherent cells CT26 (a model mouse fibroblast cell line for testing immunotherapy protocols and in studies on the host immune response) when compared to the other two treatments: the standard msEP alone and a combination of msEP with nuclear entry promotion agent, as shown in Fig. 2. More cells express green fluorescence protein (GFP) for “nsEP+msEP” samples than the other two cases (Fig. 2a). Their difference on transgene expression was further quantified by flow cytometry measurement, with the mean GFP expression level in “nsEP+msEP” samples about 3 folds and 2 folds higher than that in “msEP alone” and “msEP+TCHD” samples, respectively (Fig. 2b). This indicates that more plasmids were successfully transfected to CT 26 cells by the “nsEP+msEP” combination treatment. The transfection efficiency or number percentage of GFP positive cells was also measured and shown in Fig. 2b as follows: “nsEP+msEP” (53.5% ± 1.0%), “TCHD + msEP” (33.3 ± 0.8%) and “msEP alone” (24.3 ± 0.1%). The combined “nsEP and msEP” treatment also demonstrates a two-fold increase on the percentage of GFP positive cells than that from the standard electroporation treatment and a 50% more than that which uses TCHD nuclear entry promotion reagent. Unlike the “TCHD + msEP” treatment in which the cell viability drops nearly 30% when compared to what in the “msEP alone” benchmark case, the “nsEP+msEP” treatment does not show obvious toxicity to CT26 cells, as shown in Fig. 2c. The enhanced transgene expression in the “nsEP+msEP” combined treatment is not accompanied by the sacrifice of cell viability.

**Figure 3:**
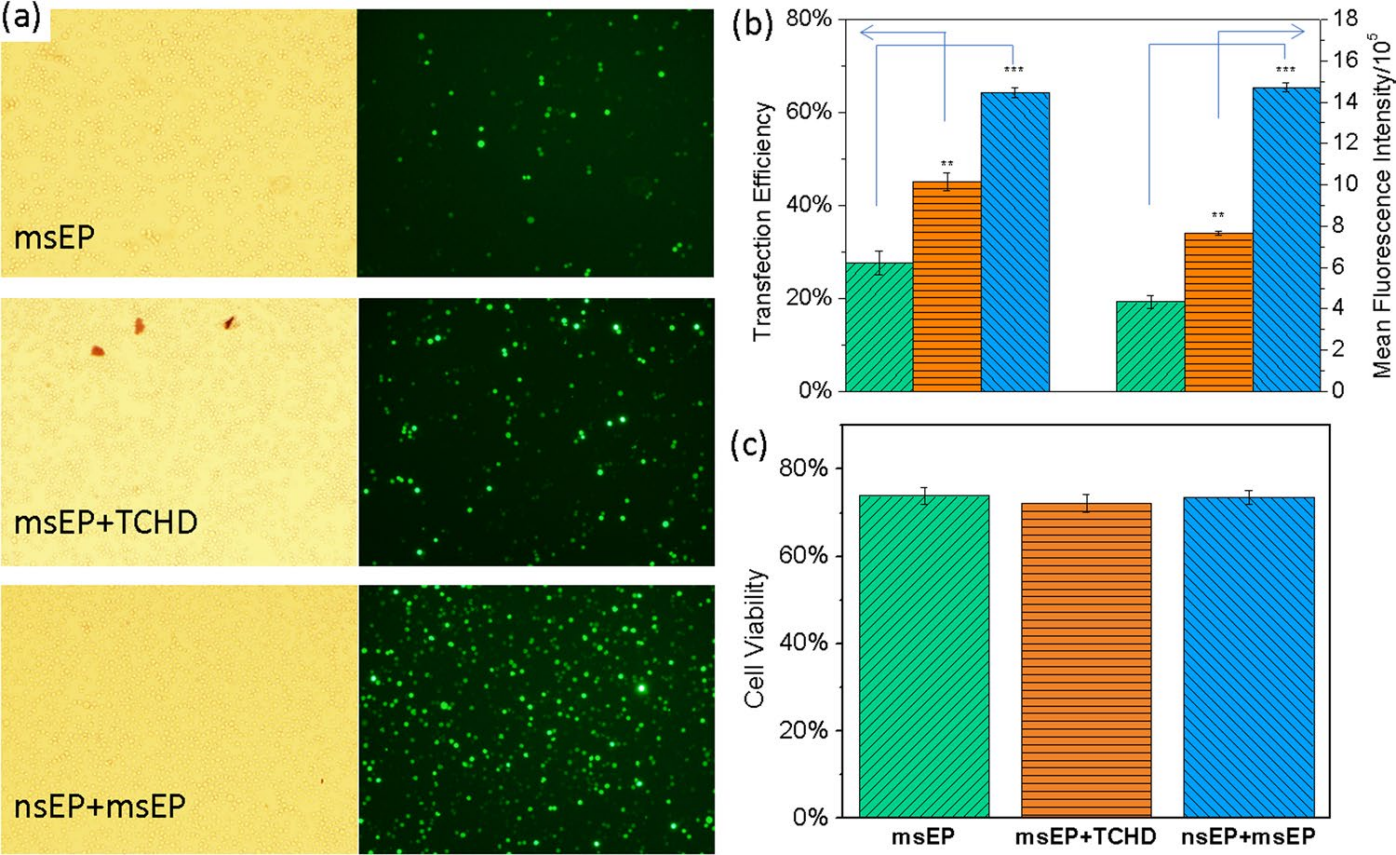
The transfection and cell viability of K562 cells 24 hrs after transfection in three difference cell transfection methods with a cell concentration of 10^7^ cells/mL. (**a**) The phase contrast (left) and fluorescent (right) images of cells treated by each method; (**b**) quantitative transgene expression presented both as transfection efficiency for the number percentage of GFP positive cells (data on the left group) and mean fluorescence intensity for average GFP dosage in individual cells (data on the right group, readings were divided by a factor of 10^5^); (**c**) cell viability by MTS assays for cells treated by each method. In panels b and c, “msEP alone”: columns with green, upward diagonal stripes; “msEP+TCHD”: columns with orange, horizontal stripes; “nsEP+msEP”: columns with blue downward diagonal stripes. Significance t-test was performed with comparison to “msEP alone” treatment (n = 3) with **P < 0.01 and ***P < 0.005.

To demonstrate its broad effectiveness, we also did the same treatments on a suspension cell line, K562 cells (a chronic myeloid leukemia cell line). As shown in Fig. 3, similar transfection enhancement is also found on K562 cells. The standard “msEP alone” treatment receives a cells transfection of 27.6% ± 2.6% while the introduction of TCHD treatment prior to msEP cell cytoplasm delivery gains a transgene expression of 45.4 ± 2.0%, or a ~18% increase (Fig. 3b). The “nsEP+msEP” combination treatment further promotes the GFP expression level another ~18% more than what is achieved in the combined “TCHD + msEP” treatment, reaching 63.1% ± 2.8% (Fig. 3b). The mean GFP expression level is also elevated to 1.8 times (“TCHD + msEP”) and 3.4 times (“nsEP+msEP”) when compared with the benchmark “msEP alone” treatment. These results clearly prove that the combination of “nsEP+msEP” treatment more effectively enhances transgene expression of GFP for both adherent and suspension cells than the standard “msEP alone” or its combination with a nuclear entry promotion reagent. Different from what happened in CT 26 cells, no significant loss of cell viability occurs for the introduction of TCHD in K562 cells (Fig. 3c). This may be attributed to their cancer cell nature and robustness to TCHD treatment. Nevertheless, significant enhancement of plasmid transfection is achieved for the “nsEP+msEP” treatment without compromising cell viability loss. It is more effective over not only the standard electroporation (i.e., “msEP”), but also many well adopted chemical transfection approaches (see Supplemental Fig. S3), considering a balance of transgene expression and cell viability as well as the probe-to-cell ratio.

### Enhancement on transgene expression kinetics

**Figure 4:**
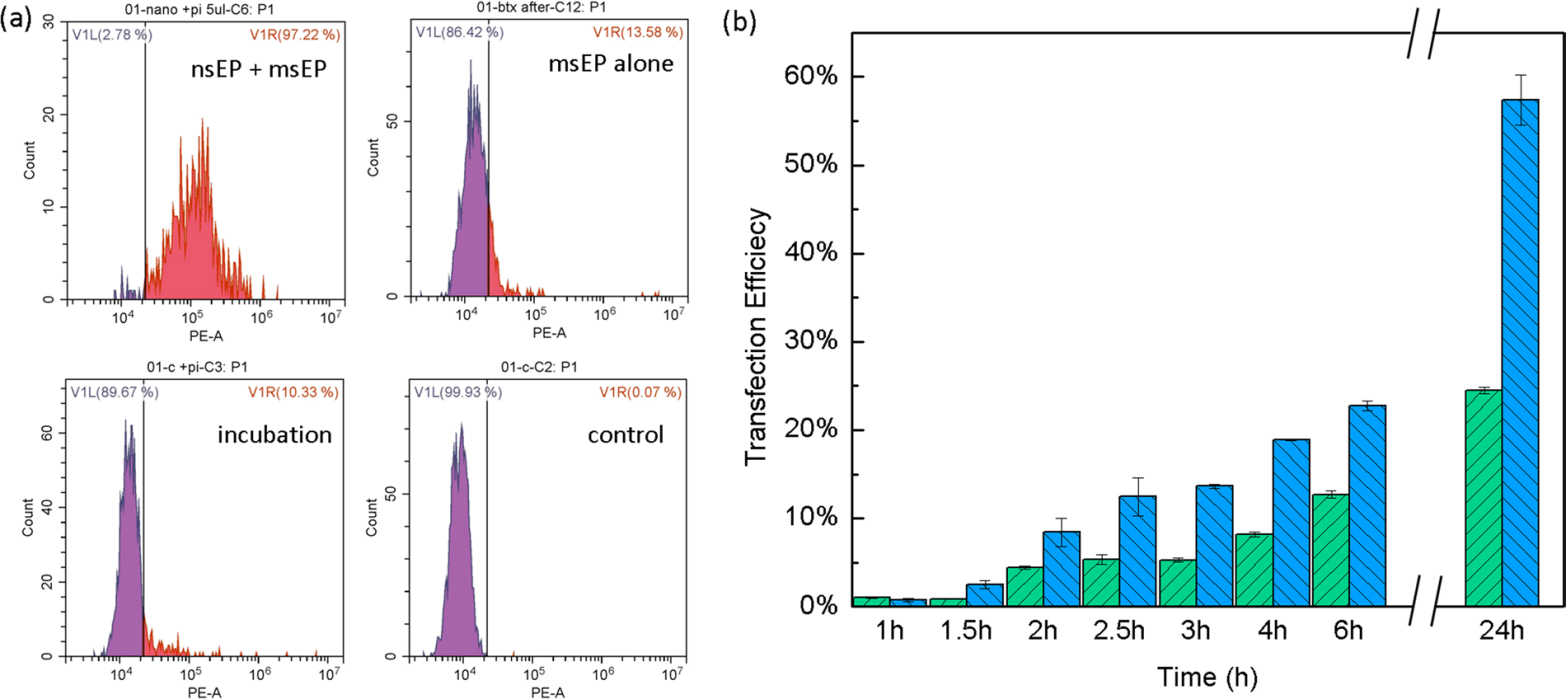
(**a**) The PI dye uptake and (**b**) transgene expression kinetics of GFP plasmid in K562 cells for nsEP+msEP combination and msEP alone treatments. The nsEP conditions: 50 kV/cm (pulse strength), 100 kHz (pulse frequency), and 600 ns (pulse duration) and the msEP condition: 550 V/cm (pulse strength), one pulse, and 10 ms (pulse duration). In panel b, “msEP alone”: columns with green, upward diagonal stripes; nsEP+msEP: columns with blue downward diagonal stripes.

As we hypothesize nsEP treatment has advantages on cell nuclear membrane disruption, the transgene expression time should be greatly shortened for plasmid DNA probes with permeable nuclear membrane and quick nuclear transport. To verify this hypothesis, we measured both the uptake of propidium iodide (PI) dye and expression kinetics the GFP plasmids using flow cytometry. As shown in Fig. 4a, the uptake of PI dyes reaches 97.2% after the “nsEP+msEP” combination treatment. In contrast, the “msEP alone” treatment gets only 13.6%, which is only slightly higher than the case for simply incubating PI dyes with the cell solution (10.3%). Considering the membrane-impermeant nature of PI dyes and the fact that fluorescence signal emits only if PI dye molecules intercalate into the major groves of double-stranded DNA molecules, these results indicate that msEP alone cannot effectively disrupt the integrity of cell nuclear membrane, though the permeable cell membrane after msEP allows PI dyes to arrive in cell cytosol. With additional nsEP treatment (i.e., for “nsEP+msEP” case), the dramatic increase of the fluorescence signal confirms that both the plasma membrane and the nuclear membrane of cells are successfully broken down to allow PI dyes to flush in and migrate all the way to cell nucleus to conjugate with DNA molecules for fluorescence emission. To further prove the effectiveness of nsEP treatment on nuclear delivery, the expression kinetics of GFP plasmids in K562 cells is also monitored by measuring the DNA expression level at various moments of the first 24 hours after the “nsEP+msEP” combination treatment. The enhancement on green fluorescent signal from GFP proteins was detected as early as 1.5 hours post treatment, as shown in Fig. 4b. By 6 hours, the GFP expression level in the “nsEP+msEP” samples is almost the same with what in the “msEP alone” treatment 24 hours post transfection. From these results, we conclude that the contribution of nsEP towards the enhancement of transfection rate is likely the result from fast and massive transport of DNA plasmid across the cell nuclear membrane so that early and efficient transcription process is initiated.

### Importance on the treatment order of nsEP and msEP

**Figure 5:**
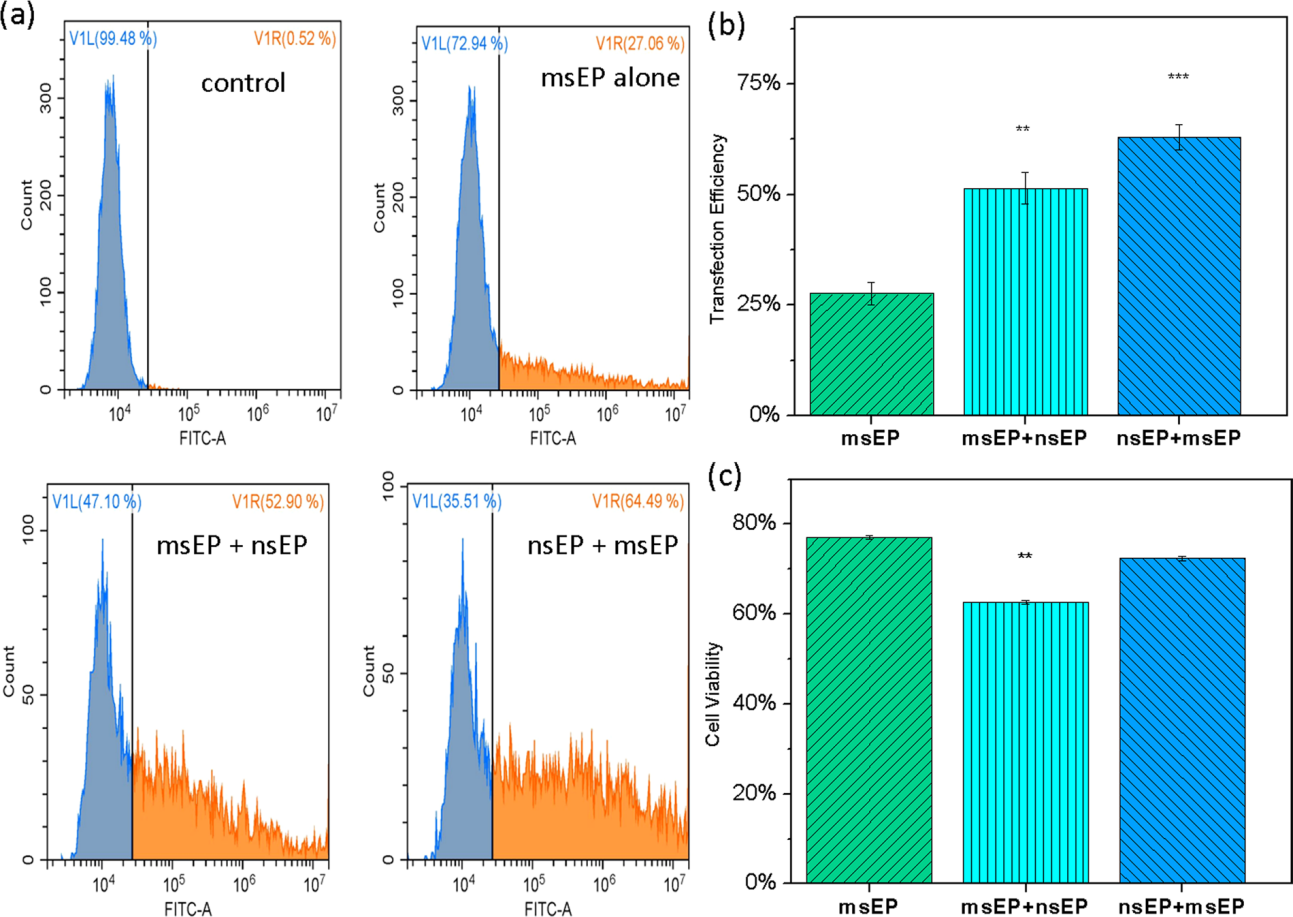
The effect of the combination order of msEP and nsEP on DNA delivery: (**a,b**) transfection efficiency and (**c**) cell viability. In panels b & c, “msEP alone”: columns with green, upward diagonal stripes; “msEP+nsEP”: columns with cyan, vertical stripes; “nsEP+msEP”: columns with blue downward diagonal stripes. Significance t-test was performed with n = 3 and **represents P < 0.01 and *** for P < 0.005 with comparison made to “msEP alone” treatments.

Although nsEP helps make the nuclear membrane more permeable, significant enhancement of gene expression would not be achieved if efficient crossing of the cell plasma membrane does not occur. We learned this from our early failed experimental efforts. In some of those early-stage experiments, the nsEP treatment was done alone under the same pulse conditions, but no successful plasmid transfection was found. This suggests that nanosecond pulses alone cannot make cell membrane permeable enough to allow effective cell uptake of plasmid DNA. In another set of experiments in which the msEP treatment was delayed one hour after the completion of nsEP treatment, we found the transgene expression enhancement effect also disappeared. These results suggest that without quick introduction of plasmid DNA in cell cytosol, the disrupted nuclear membrane might recover its integration before DNA molecules arrive there so that the nsEP treatment, which has the benefit of nuclear permeability enhancement, would be done in vain. In a different case, we switched the execution order of nsEP and msEP treatment combination. When msEP is done before nsEP in the combined treatment (designated as “msEP+nsEP”), the transgene expression of GFP is not as high as its opposite combination order (“nsEP+msEP). As shown in Fig. 5a,b, the GFP expression for the “msEP+nsEP” treatment receives a transfection efficiency of 51.4 ± 3.6%, about 12% lower than what with the combination order as “nsEP+msEP” (63.1% ± 2.8%) at the same pulse conditions. Moreover, the cell viability of the “msEP+nsEP” treatment also drops about 10% when compared to what in the “nsEP+msEP” cases (Fig. 5c). This suggests that having already disrupted cell membrane makes further polarization of cell nuclear membrane less effective. The followed high-frequency nanosecond pulses cause more loss of ions or molecules from the treated cells than what happened in the “msEP alone” treatment. Their damage to cells is so severe that the promotion to the nuclear delivery of DNA is largely offset by the slow recovery of cellular membrane and some metabolic functions of the treated cells, resulting in their low survival rate. This is probably one major reason that undesired low transfection efficiency and/or cell viability were received in other nanosecond pulse treatments^18^.

### Contribution of microfluidics on electroporation enhancement

**Figure 6:**
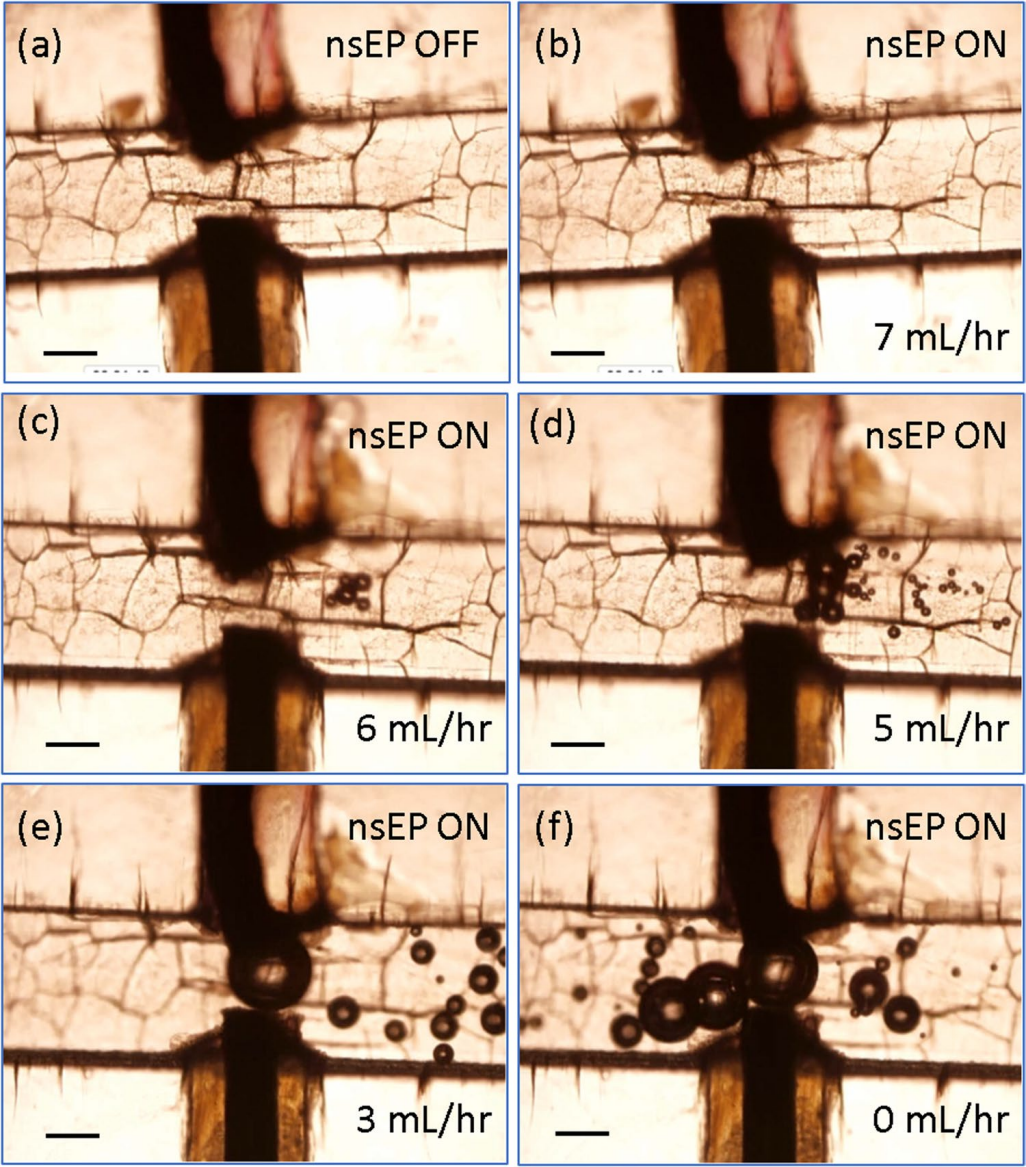
Gas bubble formation and evolution dynamics in microfluidic nsEP treatment at various flow rates. Photos of microfluidic device without nsEP treatment (**a**) and with nsEP treatment of a K562 cell solution flowing at 7 ml/hr (**b**), 6 ml/hr (**c**), 5 ml/hr (**d**), 3 ml/hr (**e**), and 0 ml/hr (**f**). The scale bars represent 100 µm. (Note: texture shown in the channel region of each image comes from winkles of scotch tape sealing the microfluidic channel).

The introduction of microfluidics during nsEP enables close patterning of Pt electrodes so that a low pulse voltage is sufficient to meet the required field strength for effective nanosecond treatment, besides its additional benefit of high throughput on cell processing. More important, we found a microfluidics configuration helps suppress the gas bubble evolution and Joule heating issues that often lead to low cell viability during nanosecond pulse treatment. As shown in Fig. 6a,b and supplemental move I, no visible gas bubbles are found near the electrodes with a 100x magnification at the regular flow rate in our nsEP treatment (7 ml/hr). However, if the cell flow is slowed down, gas bubbles are clearly observed to appear continuously near the electrode surface when the nanosecond pulse signal is turned on (Fig. 6c–f and supplemental movies II-V). The generated gas bubbles grow quickly on the electrode surface while are eventually taken away by flow to downstream when getting too big. The slower the liquid flow, the bigger those gas bubbles are. This suggests that the suppression of electrohydrolysis and Joule heating phenomena by this microfluidic nsEP system comes from the quick takeaway of bubbles from their generation spots (the Pt electrodes) so that the associated gas bubble evolution issues are minimized. As the result, high cell viability and better enhancement of transgene expression (i.e., more transfected cells survived the electroporation treatment with normal metabolism) are achieved simultaneously with this microfluidic “nsEP+msEP” treatment.

## Discussions

The generation of electric pulses with both high-voltage field strength and a mixture duration of millisecond and nanosecond duration is not trivial. Severe signal entanglement and profile distortion often occur. Two design features are applied in our system to avoid these issues. First, we separate the nanosecond pulse generator and high-voltage supply units into two different electric circuits, which are bridged by a MOSFET switch. The frequency (HZ) and duration (ns) of the nanosecond pulses are decided by the pulse signal generator while the high-voltage electric field imposed on cells is supplied by the pre-charged capacitor through the high-voltage supply circuit. The decoupling of the pulse voltage and duration (and frequency) in our nsEP treatment helps maintain appropriate pulse profile when some other pulse parameters are changed. Together with the Pt wire electrode pair, nanosecond pulses in our nsEP system have ultra-short responding time, well-defined signal profile, and stable pulse voltage. For example, when the pulse width or amplitude is changed, the waveform (amplitude or width) of those nanosecond pulses, including the primary rectangular part and the reflection tail, is well retained. Another design we adopted to avoid signal entanglement lies on the separation of the nsEP and msEP treatments with different devices. A microfluidics-based nsEP treatment is followed by a cuvette-based standard msEP electroporation. Such configuration separation allows the complete decoupling of the two types of pulse signals in nsEP and msEP treatments. The pulse conditions for each treatment can be tuned separately without interactions from the other, making the comparison and optimization of electroporation protocols easier and more reliable.

Besides quick responding time, the proximity placement of the two Pt wire electrodes on the side wall of the microfluidic channel further allows relatively low applied voltage or energy dose and long pulse duration in our nsEP treatment. This helps collapse the nuclear membrane more effectively without much cell toxicity, unlike what is observed in irreversible electroporation with ultrashort pulses. Moreover, the introduction of a microfluidic configuration in the nsEP treatment mitigates electrohydrolysis-induced side effects such as gas bubble evolution and Joule heating, another major negative impact of other nanosecond pulse treatments on permanent damage to cells and/or consequent cell apoptosis. Gas bubble formation is common in electroporation, contributed by water electrohydrolysis. With the presence of gas bubbles, they disturb the instantaneous local electric field, making the followed pulse disruption on neighbor cells much less effective. Moreover, the accompanied Joule heating further elicits bubble’s growing, rising, and bursting^22^. Since many cells are trapped around gas bubbles, such bubble evolution dynamics causes further detrimental damage to cells, varying from impact on cell physiological response to complete cell lysis. In our microfluidic nsEP treatment step, gas bubbles were generated on the electrode surface like in most studies employing electroporation. However, the generated gas bubbles were quickly taken to downstream by flow, away from the Joule heating source (i.e., near Pt electrodes) before growing too big. Such suppression of gas bubble evolution is one key to the success of this microfluidic “nsEP+msEP” combination treatment. Similar gas bubble evolution dynamics was also found in our early studies on cuvette-based standard msEP treatment^24^. With still fluid in a cuvette which stands straight up, gas bubbles are generated, further grow, rise, aggregate, and burst when arriving at the air-liquid interface. A strong convection of liquid caused by gas bubble dynamics during pulsation period not only traps and damages cells (particularly when burst), but also greatly affects the local electric field of the followed pulses. Although the gas bubble issues vary with the pulse conditions such as pulse type, strength, and duration, it is probably another cause of the disappointing transgene expression outcomes in some early nanosecond pulse studies employing cuvettes in their entire treatments^18^. As this issue is largely suppressed in this microfluidic “nsEP+msEP” treatment, it is not surprising that better enhancement of transgene expression and high cell viability are achieved simultaneously.

## Conclusions

In summary, we developed a new microfluidic electroporation approach with a combination of nanosecond and millisecond pulses to enhance plasmid DNA transfection without sacrifice on cell viability. For adherent cells CT26 and suspension cells K562, transgene expression of GFP plasmid is more than doubled at the same cell viability levels as in standard msEP electroporation. Promotion on nuclear entry by nanosecond pulses and the suppression of gas bubbles with microfluidic operation are believed to be the two major contributions by this new system on the transfection enhancement. Having nsEP first and msEP followed immediately is found to work more effectively than other cases with either the opposite operation order or having a long waiting period between the two types of pulse treatments. This new microfluidic nsEP and msEP combination treatment not only facilitates plasmid delivery and expression, but also eliminates the electrohydrolysis-induced apoptosis and low cell viability issues commonly tied with nanosecond pulse treatments. Its success may benefit many biology studies and clinical practice in understanding gene functions, controlling cellular signals, screening drugs, and applying cell-based therapeutic technologies.

## Methods

### Materials and reagents

DNA plasmids with pMaxGFP reporter genes were purchased from Lonza, Inc. All other cell culture reagents were purchased from Life Technologies (Carlsbad, CA) and chemicals were purchased from Sigma-Aldrich unless specified.

### Cell culture

A human chronic myelogenous leukemia (CML) cell line, K562 (ATCC, CCL-243) and a murine colorectal carcinoma cell line from a BALB/c mouse, CT26.WT cells (ATCC, CRL-2638), were used in electroporation experiment as the representative of suspension cells and adherent cells, respectively. These cells were cultured in RPMI 1640 medium (supplemented with 10% FBS, 1% L-Glutamine and Penicillin/streptomycin). All cultures were maintained at 37 °C with 5% CO_2_ and 100% relative humidity.

### Nano-pulse Electroporation device fabrication

A home-made circuit board was designed to generate the nanosecond pulses with different parameters of voltage (V), frequency (HZ), and duration (ns). The circuit board includes three separated units which are connected with an n-channel enhanced radio frequency metal-oxide-semiconductor field-effect transistor (MOSFET), as shown in Fig. 1a. The pulse generation unit includes a low-voltage pulse generator (Agilent 33220 A), a resistor load carrier, and a MOSFET switch. The power supply unit has a high-voltage DC power supply (KIKUSUI PMC250-0.25 A), two resistors, and a capacitor (shared with the electroporation unit). The electroporation unit includes a capacitor (shared with the power supply unit), a resistor that allocates pulse load, and the electroporation device (either cuvette-type from standard electroporation or our home-made microfluidic-type one). In this nsEP circuit, the power supply pre-charges the capacitor which is used to store energy (when the MOSFET switch is OFF) and supplies the needed high-voltage pulses on cells (when the MOSFET switch is ON). The duration and frequency of these pulses is decided by the rectangular pulse signal generated from Agilent 33220 A. By periodically supplying the threshold gate voltage of the MOSFET switch, the pulse generator triggers the turn-on moment and duration of the MOSFET switch in each pulse. The MOSFET switch manages the open and closure the electroporation circuit so that the pre-charged capacitor from the electroporation unit discharges accordingly to allow the high-voltage pulse to pass through the electroporation device with designed nanosecond width and number. An oscilloscope is used to monitor the instant nanosecond pulse profile to ensure its appropriateness. Depending on the dimensions of the electroporation device (e.g., a gap size of a standard electroporation cuvette or Pt wire electrode pair), proper resistors (R3 in Fig. 1a) will be chosen in the electroporation circuit so that its resistance is comparable to what the cell sample load has for best electroporation performance.

A microfluidic channel (500-µm wide, 300-μm deep, and 5-cm long) was milled on a piece of poly (methyl methacrylate) or PMMA board by a computer numeric control (CNC) machine. A piece of platinum wire (0.25 mm in diameter) was pre-embedded in the PMMA board, perpendicular to the flow channel direction. It was then cut into two separated wire electrodes during the micromilling process with a gap size of 200 µm or longer (the actual separation distance between the Pt electrode pair is measured after device fabrication), as shown in Fig. 1b. Copper wire cables were then connected the two Pt electrodes with the nanopulse electroporation circuit. To monitor gas bubble issues in nsEP, the nsEP microfluidic device was mounted on the stage of an inverted microscopy (Olympus IX71) and videos were taken to record the gas bubble evolution at different flow rates.

### Electroporation setup and process

Cells were first centrifuged and re-suspended in fresh OPTI-MEM I (a serum free medium) at a density of 1 × 10^7^ cells/mL. Plasmid DNA (pMaxGFP) of 10 µg was then added in each electroporation sample (100 µL). These samples are first loaded into the microfluidic channel at a flow rate of 7 ml/hr to receive nsEP treatment. Nanosecond pulses with an electrical field of ~25–70 kV/cm, a pulse width of 400–800 ns, and a pulse frequency of 1–400 kHz were applied across the two Pt wire electrodes. The cell solution experienced the nsEP treatment was continuously collected in a traditional electroporation cuvette downstream (with the parallel electrodes separated by 1–4 mm) to receive immediate standard electroporation (550 V/cm, one 10-ms pulse with a rectangular pulse profile). After the msEP treatment, cells were transferred to 12-well plates and cultured for 24 hours at 37 °C with 5% CO_2_ and 100% relative humidity prior to further analysis.

### Transfection efficiency

The expression of pMaxGFP plasmids is evaluated both qualitatively by visualizing cells with green fluorescence within some representative areas under an inverted fluorescence microscope (Olympus, Japan) and quantitatively using flow cytometry (CytoFLEX PN B49006AE) with a laser wavelength of 495–519 nm. The results were analyzed with Cytexpert software and 10,000 events were counted for each sample. The transfection efficiency of pMaxGFP is defined as the number of cells emitting fluorescence signal to the total number of counted cells in a sample (gated fluorescence signal of GFP). The GFP expression level is quantified as the mean fluorescence intensity of the whole counted cell population, which is correlated to the average dosage of plasmids delivered in individual cells. Their combination reflects a comprehensive picture of plasmid delivery and expression level in treated cells. In GFP plasmid expression kinetics experiments, transfected cells were distributed in 24-well culture plates and collected for flow cytometry measurements at various defined moments (1 hr, 1.5 hrs, 2 hours, 2.5 hours, 3 hours, 4 hours, 6 hours, and 24 hours) post transfection. In PI dye uptake experiments, 1 µl PI dye solution (1 µg/µl) was added to the cell solution (100 µl) and incubated for 20 minutes before further analysis or treatments.

### Cell viability

MTS assays were used to evaluate cell viability. Briefly, 100 µL cells 24 hr post electroporation from each sample were harvested from their culturing cells and transferred to a 96-well plate. CellTiter 96 AQueous One solution (Promega, Madison, WI) of 20 µL was added to each well and all samples were incubated at 37 °C for 1 hr. The absorbance signal of color formazan product from the reduction of MTS tetrazolium compound by dehydrogenase enzymes in metabolically active cells is measured at 492 nm on an automated plate reader (Elx 800, Biotek, VT) to reflect the number of viable cells present in each sample under defined conditions. The cell viability is calculated as the absorbance signal ratio of an electroporated cell sample to what from the negative control cell sample, after extracting the absorbance background from the media.

### Statistics

All numerical data were generated from at least three independent experiments and represented as the mean ± standard deviation (SD) for each experimental condition unless otherwise indicated. Significance analysis was performed with two-tailed *t*-test.

## Supplementary information

Supplemental materials.

Supp Movie 1 (7 ml/hr).

Supp Movie 2 (6 ml/hr).

Supp Movie 3 (5 ml/hr).

Supp Movie 4 (4 ml/hr).

Supp Movie 5 (3 ml/hr).

Supp Movie 6 (0 ml/hr).

## Data Availability

All experimental data of this study are stored and available from the corresponding author S.W. for sharing upon request unless it may lead to the copyright violations from the publishers or other organizations.
