# Supplementary material for: Microfluidic Electroporation Coupling Pulses of Nanoseconds and Milliseconds to Facilitate Rapid Uptake and Enhanced Expression of DNA in Cell Therapy
Source: Sci Rep. 2020 Apr 8;10:6061. doi: 10.1038/s41598-020-63172-8 (PMC7142113; doi:10.1038/s41598-020-63172-8)
Supplement: Supplementary file 1 — Supplemental materials. [file 41598_2020_63172_MOESM1_ESM.pdf]

Microfluidic Electroporation Coupling Pulses of Nanoseconds and Milliseconds to Facilitate Rapid Uptake and Enhanced Expression of DNA in Cell Therapy

An-Yi Chang[a, b, d,#], Xuan Liu[c,d,#], Hong Tian[b], Liping Hua[b,d], Zhaogang Yang [e], and Shengnian Wang[a, b,c, d]*

[a] *Chemical Engineering,* [b] *Institute for Micromanufacturing ,*[c] *Macromolecular and Nanotechnology,* [d]*Center for Biomedical Engineering and Rehabilitations, Louisiana Tech University, PO Box 10137, Ruston, LA, 71272 USA*
[e]*Department of Radiation Oncology, The University of Texas Southwestern Medical Center, Dallas, TX 75390, USA*

# These authors contribute equally.
∗ Corresponding author:
Tel: +1 (318)257-5125; Fax: +1 (318) 257-5104; E-mail: swang@latech.edu

Content :

Supplemental Figure S1 on the pulse condition optimization for nsEP treatment on CT 26 cells and K562 cells.

Supplemental Figure S2 on comparison of transfection performance of “nsEP+msEP” electroporation treatment with various cell number or concentration in a 100 μL electroporation solution.

Supplemental Figure S3 on comparison of transfection performance of “nsEP+msEP” electroporation treatment with other standard transfection methods.

Supplemental movies on gas bubble formation and evolution dynamics in microfluidic nsEP treatment of a K562 cell solution at various flow rates: (a) 7 ml/hr, (b) 6 ml/hr, (c) 5 ml/hr, (d) 4 ml/hr, (e) 3 ml/hr, and (e) 0 ml/hr. The scale bars represent 100 μm. (Note: texture shown in the channel region of each image comes from winkles of scotch tape sealing the microfluidic channel).

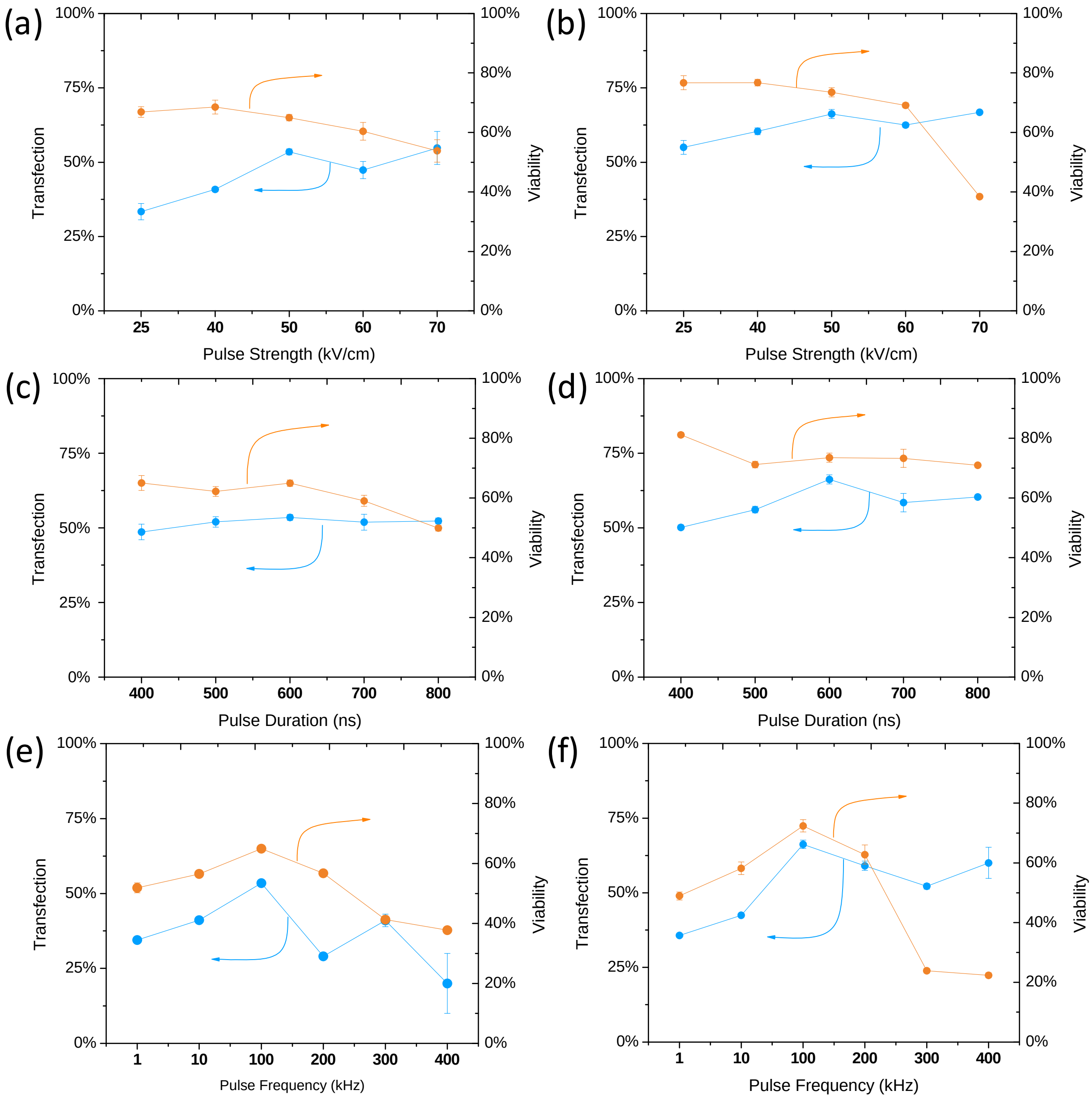

Supplemental Figure S1. The pulse condition optimization for nsEP treatment on CT 26 cells (a-c) and K562 cells (d-f) based on a balance of transfection and cells viability. The transfection and cell viability for nsEP with pulses of different electric voltages (0.5-1.4kV) under a fixed pulse frequency (100 kHz) and duration (600ns) (a, c), different pulse duration (400-800 ns) under a fixed pulse voltage (1.0 kV) and frequency (100 kHz) (b, e), and different frequencies (1-400 kHz) under a fixed pulse duration (600ns) and voltage (1.0 kV).

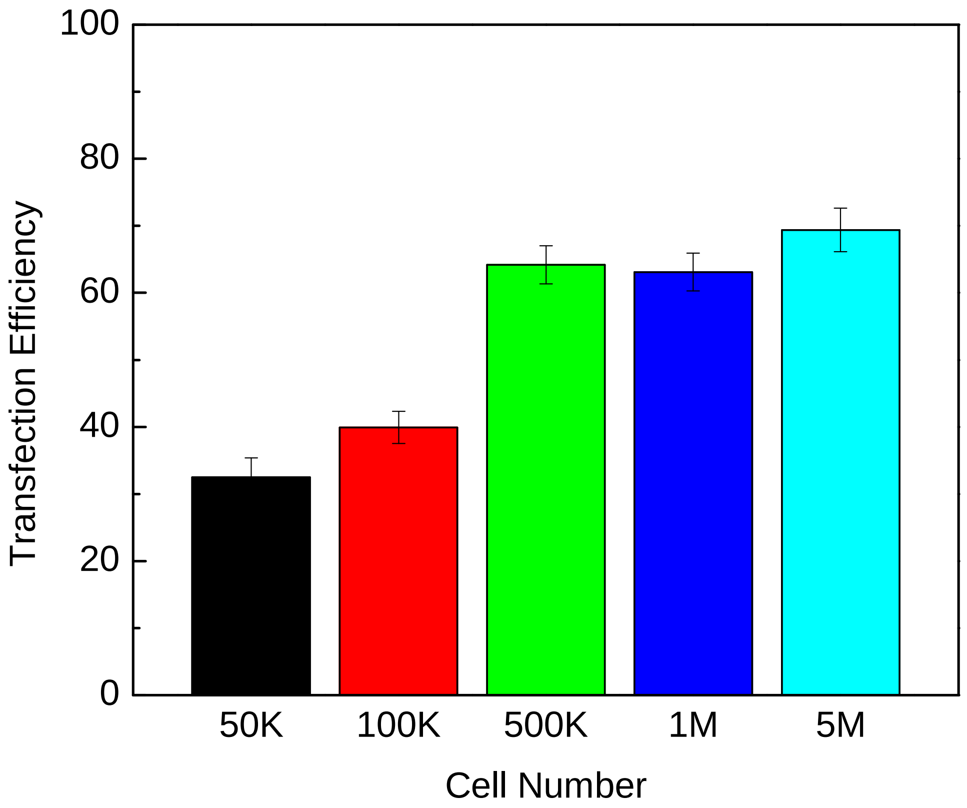

Supplemental Figure S2. Cell number effect on transfection performance of “nsEP+msEP” electroporation treatment in K562 cells. Transgene expression is presented as GFP positive cell number percentage in each cell sample. The volume of the electroporation solution is 100 μL and the GFP plasmid dosage to cell number ratio is fixed as 10 μg pMaxGFP DNA/10^6 cells.

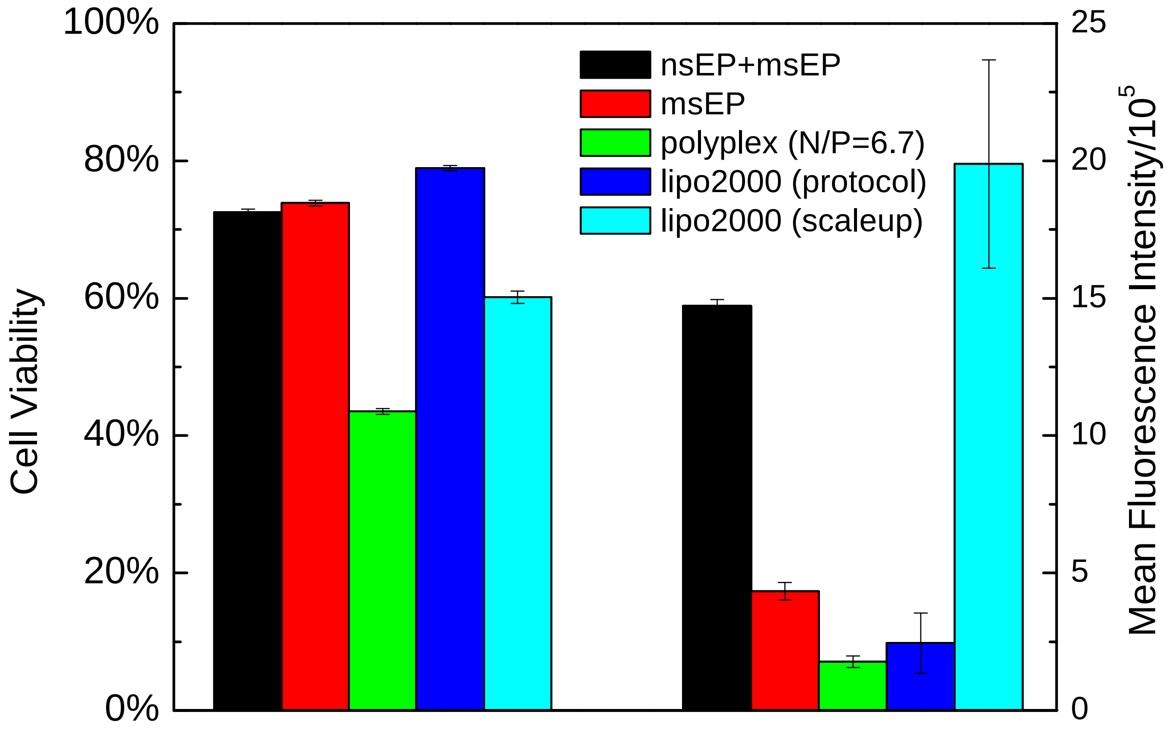

Supplemental Figure S3. Comparison of transfection performance of "nsEP+msEP" electroporation treatment with other standard transfection methods in K562 cells. Standard electroporation is the same as the case designated as "msEP alone" in this paper; "polyplex (N/P=6.7)" represents polyplex transfection cases with samples prepared by vortex mixing polyethylenimine (PEI, branched with MW=25K from Sigma-Aldrich) and pmaxGFP plasmid at a N/P ratio of 6.7 (the molar ratio of positively charged amine groups on PEI with negatively charged phosphate groups on DNA); "lipo2000 (protocol)" represents lipofecamine 2000 (from Life Technologies) transfection cases following the vendor's protocol (1 μg plasmid, 2 μl lipofectamine 2000 for 4×10^4 cells); "lipo2000 (scale-up)" represents lipofectamine 2000 transfection cases having probe dosage scaled up according to the cell numbers that is used in our electroporation treatment (25 μg plasmid, 50 μl lipofectamine 2000 for 1×10^6 cells). The transfection is shown as mean fluorescence intensity measurement by flow cytometry (columns on the right-side group) and the cell viability is quantified via MTS assay (columns on the left-side group), with three independent experiments.
